# Supplementary material for: Thermodynamic efficiency in dissipative chemistry
Source: Nat Commun. 2019 Aug 27;10:3865. doi: 10.1038/s41467-019-11676-x (PMC6711991; doi:10.1038/s41467-019-11676-x)
Supplement: Supplementary file 1 — Supplementary Information [file 41467_2019_11676_MOESM1_ESM.pdf]

Supplementary Information for
"Thermodynamic Efficiency in Dissipative Chemistry"

Emanuele Penocchio,[1] Riccardo Rao,[1,2] and Massimiliano Esposito[1,*]

[1]*Complex Systems and Statistical Mechanics, Physics and Materials Science Research Unit, University of Luxembourg, L-1511 Luxembourg, G.D. Luxembourg*

[2]Present Address: *The Simons Center for Systems Biology, School of Natural Sciences, Institute for Advanced Study, Princeton, 08540 New Jersey, U.S.A.*

CONTENTS

Supplementary Note 1: Details on Energy Storage 1
a. Dynamics 1
b. Thermodynamics 2
c. Cycles & kinetic symmetry 5
d. Parameters 6

Supplementary Note 2: Details on Driven Synthesis 7
a. Dynamics 7
b. Thermodynamics 7
c. Plots of $-\dot{\mathcal{W}}_{\text{ext}}$ and $\dot{\mathcal{W}}_{\text{fuel}}$ 8
d. Linear Regime 8

Supplementary References 11

SUPPLEMENTARY NOTE 1: DETAILS ON ENERGY STORAGE

a. Dynamics

The evolution in time of the concentrations of the species M, M^*, A_2^*, and A_2 is ruled by the rate equations

$$\mathrm{d}_t \underbrace{\begin{pmatrix} [\mathrm{M}] \\ [\mathrm{M}^*] \\ [\mathrm{A}_2^*] \\ [\mathrm{A}_2] \end{pmatrix}}_{[\boldsymbol{X}]} = \underbrace{\begin{pmatrix} -1 & -1 & 0 & 0 & 0 & 2 \\ 1 & 1 & -2 & 0 & 0 & 0 \\ 0 & 0 & 1 & -1 & -1 & 0 \\ 0 & 0 & 0 & 1 & 1 & -1 \end{pmatrix}}_{\mathbb{S}^{\mathrm{X}}} \cdot \underbrace{\begin{pmatrix} k_{+1\mathrm{F}}[\mathrm{F}][\mathrm{M}] - k_{-1\mathrm{F}}[\mathrm{M}^*] \\ k_{+1\mathrm{W}}[\mathrm{W}][\mathrm{M}] - k_{-1\mathrm{W}}[\mathrm{M}^*] \\ k_{+2}[\mathrm{M}^*]^2 - k_{-2}[\mathrm{A}_2^*] \\ k_{+3\mathrm{F}}[\mathrm{A}_2^*] - k_{-1\mathrm{F}}[\mathrm{A}_2][\mathrm{F}]^2 \\ k_{+3\mathrm{W}}[\mathrm{A}_2^*] - k_{-1\mathrm{W}}[\mathrm{A}_2][\mathrm{W}]^2 \\ k_{+4}[\mathrm{A}_2] - k_{-4}[\mathrm{M}]^2 \end{pmatrix}}_{\boldsymbol{J} = \boldsymbol{J}_+ - \boldsymbol{J}_-}, \tag{1}$$

* massimiliano.esposito@uni.lu

where [F] and [W] are the concentrations of fuel and waste species. Since these latter are externally kept constant by the chemostats, the balance equations for their concentrations read

$$\mathbf{0} = \mathrm{d}_t \underbrace{\begin{pmatrix}[\mathrm{F}]\\ [\mathrm{W}]\end{pmatrix}}_{[Y]} = \underbrace{\begin{pmatrix}-1 & 0 & 0 & 2 & 0 & 0\\ 0 & -1 & 0 & 0 & 2 & 0\end{pmatrix}}_{\mathbb{S}^{\mathrm{Y}}} \cdot \underbrace{\begin{pmatrix} k_{+1\mathrm{F}}[\mathrm{F}][\mathrm{M}] - k_{-1\mathrm{F}}[\mathrm{M}^*]\\ k_{+1\mathrm{W}}[\mathrm{W}][\mathrm{M}] - k_{-1\mathrm{W}}[\mathrm{M}^*]\\ k_{+2}[\mathrm{M}^*]^2 - k_{-2}[\mathrm{A}_2^*]\\ k_{+3\mathrm{F}}[\mathrm{A}_2^*] - k_{-1\mathrm{F}}[\mathrm{A}_2][\mathrm{F}]^2\\ k_{+3\mathrm{W}}[\mathrm{A}_2^*] - k_{-1\mathrm{W}}[\mathrm{A}_2][\mathrm{W}]^2\\ k_{+4}[\mathrm{A}_2] - k_{-4}[\mathrm{M}]^2 \end{pmatrix}}_{\boldsymbol{J} = \boldsymbol{J}_+ - \boldsymbol{J}_-} + \underbrace{\begin{pmatrix}I_{\mathrm{F}}\\ I_{\mathrm{W}}\end{pmatrix}}_{\boldsymbol{I}}, \tag{2}$$

with I_F and I_W denoting the external currents of fuel and waste flowing from the chemostats. We denote by $X = \mathrm{M}, \mathrm{M}^*, \mathrm{A}_2, \mathrm{A}_2^*$ the internal species, by $Y = \mathrm{F}, \mathrm{W}$ the chemostatted ones, and label by $\rho = 1\mathrm{F}, 1\mathrm{W}, 2, 3\mathrm{F}, 3\mathrm{W}, 4$ the reactions.

b. Thermodynamics

We consider an isothermal, isobaric, and well-stirred ideal dilute solution containing species undergoing elementary reactions. Each species is thermodynamically characterized by chemical potentials of the form

$$\mu_X = \mu_X^\circ + RT \ln \frac{[X]}{[0]}\,, \quad \mu_Y = \mu_Y^\circ + RT \ln \frac{[Y]}{[0]}\,, \tag{3}$$

where μ_X° and μ_Y° are standard-state chemical potentials and [0] is the standard-state concentration.

Dynamics and thermodynamics are related via the hypothesis of local detailed balance, which relates the ratio of rate constants to the differences of standard-state chemical potentials along reactions

$$RT \ln \frac{k_{+\rho}}{k_{-\rho}} = -\sum_X \mu_X^\circ \mathbb{S}_\rho^X - \sum_Y \mu_Y^\circ \mathbb{S}_\rho^Y\,. \tag{4}$$

At equilibrium, the thermodynamic forces driving each reaction, also called affinities, vanish

$$A_\rho^{\mathrm{eq}} = -\textstyle\sum_X \mu_X^{\mathrm{eq}} \mathbb{S}_\rho^X - \sum_Y \mu_Y^{\mathrm{eq}} \mathbb{S}_\rho^Y = 0\,, \tag{5}$$

as well as all reaction currents

$$J_\rho^{\mathrm{eq}} = J_{+\rho}^{\mathrm{eq}} - J_{-\rho}^{\mathrm{eq}} = 0\,. \tag{6}$$

The dissipation of the process is captured by the entropy production (EP) rate

$$T\dot{\Sigma} = RT \sum_\rho J_\rho \ln \frac{J_{+\rho}}{J_{-\rho}} \geq 0\,, \tag{7}$$

which also vanishes at equilibrium. Using the rate equations and the local detailed balance, Supplementary Equation 4, one can rewrite this quantity as

$$T\dot{\Sigma} = -\mathrm{d}_t G + \dot{\mathcal{W}}_{\mathrm{chem}}\,, \tag{8}$$

where

$$G = \textstyle\sum_X [X] \left(\mu_X - RT\right) + \sum_Y [Y] \left(\mu_Y - RT\right) \tag{9}$$

is the Gibbs free energy, while

$$\dot{\mathcal{W}}_{\text{chem}} = \sum_Y \mu_Y I_Y = \mu_F I_F + \mu_W I_W \tag{10}$$

is the chemical work per unit time exchanged with the chemostats.

One can also show that if the CRN were closed (fuel and waste not chemostatted) it would relax to equilibrium by minimizing G [1]. Fuel and waste are however chemostatted and we need to identify the conditions for equilibrium in the open CRN. To do so we preliminary identify the topological properties of the network.

The stoichiometric matrix $\mathbb{S} \equiv (\mathbb{S}^X, \mathbb{S}^Y)^T$ (see Supplementary Equations 1 and 2) encodes the topological properties of the CRN. We can access these properties by determining its cokernel, which is spanned by

$$\boldsymbol{\ell}_M = \overset{\begin{matrix}\mathrm{M} & \mathrm{M}^* & \mathrm{A}_2^* & \mathrm{A}_2 & \mathrm{F} & \mathrm{W}\end{matrix}}{\begin{pmatrix} 1 & 1 & 2 & 2 & 0 & 0 \end{pmatrix}}, \tag{11}$$

$$\boldsymbol{\ell}_W = \overset{\begin{matrix}\mathrm{M} & \mathrm{M}^* & \mathrm{A}_2^* & \mathrm{A}_2 & \mathrm{F} & \mathrm{W}\end{matrix}}{\begin{pmatrix} 0 & 1 & 2 & 0 & 1 & 1 \end{pmatrix}}. \tag{12}$$

The first of these vectors identifies a conserved quantity

$$L_M = \boldsymbol{\ell}_L \cdot \begin{pmatrix} [\boldsymbol{X}] \\ [\boldsymbol{Y}] \end{pmatrix} = [\mathrm{M}] + [\mathrm{M}^*] + 2[\mathrm{A}_2^*] + 2[\mathrm{A}_2], \qquad \mathrm{d}_t L_M = 0 \tag{13}$$

which is proved using the rate equations Supplementary Equation 1 and Supplementary Equation 2.

The second vector identifies what we call a broken conserved quantity

$$L_W = \boldsymbol{\ell}_W \cdot \begin{pmatrix} [\boldsymbol{X}] \\ [\boldsymbol{Y}] \end{pmatrix} = [\mathrm{M}^*] + 2[\mathrm{A}_2^*] + [\mathrm{F}] + [\mathrm{W}]. \tag{14}$$

Using again the rate equations, it can be shown that

$$\mathrm{d}_t L_W := I_F + I_W. \tag{15}$$

Namely, L_W changes only due to the exchange of fuel and waste with the chemostats. If the CRN were closed, L_W would be constant. Using Supplementary Equation 15, we can rewrite the entropy production in Supplementary Equation 8 as

$$T\dot{\Sigma} = -\mathrm{d}_t \mathcal{G} + \dot{\mathcal{W}}_{\text{fuel}}, \tag{16}$$

where

$$\begin{aligned} \mathcal{G} &= \sum_X [X](\mu_X - RT) + \sum_Y [Y](\mu_Y - RT) - \mu_W L_W \\ &= [\mathrm{M}]\mu_{\mathrm{M}} + [\mathrm{A}_2]\mu_{\mathrm{A}_2} + [\mathrm{M}^*](\mu_{\mathrm{M}^*} - \mu_W) + [\mathrm{A}_2^*]\left(\mu_{\mathrm{A}_2^*} - 2\mu_W\right) + [\mathrm{F}](\mu_F - \mu_W) + \\ &\quad - RT\left([\mathrm{M}] + [\mathrm{A}_2] + [\mathrm{M}^*] + [\mathrm{A}_2^*] + [\mathrm{F}] + [\mathrm{W}]\right) \end{aligned} \tag{17}$$

is a semigrand Gibbs potential, and

$$\dot{\mathcal{W}}_{\text{fuel}} := I_F(\mu_F - \mu_W). \tag{18}$$

is the fueling chemical work per unit of time (*i.e.*, the fueling power). The derivation of Supplementary Equation 18 for an arbitrary CRN is discussed in Supplementary References [1], [2] and [3].

If $\mu_F = \mu_W$, Supplementary Equation 16 shows that $\mathcal{G}$ is a monotonically decreasing function in time, given that $T\dot{\Sigma} \geq 0$. Its minimum value — *i.e.*, the equilibrium value — under the constraint given by

the conservation law (Supplementary Equation 13) is found by minimizing the function $\Lambda = \mathcal{G} - \lambda L_{\mathrm{M}}$, where λ is the Lagrange multiplier corresponding to L_{M}. The equilibrium concentrations thus satisfy the following conditions

$$\begin{aligned} 0 &= \left.\frac{\mathrm{d}\Lambda}{\mathrm{d}[\mathrm{M}]}\right|_{\mathrm{eq}} = \mu_{\mathrm{M}}^{\mathrm{eq}} - \lambda = \mu_{\mathrm{M}}^{\circ} + RT\ln[\mathrm{M}]_{\mathrm{eq}} - \lambda\,, \\ 0 &= \left.\frac{\mathrm{d}\Lambda}{\mathrm{d}[\mathrm{A}_2]}\right|_{\mathrm{eq}} = \mu_{\mathrm{A}_2}^{\mathrm{eq}} - 2\lambda = \mu_{\mathrm{A}_2}^{\circ} + RT\ln[\mathrm{A}_2]_{\mathrm{eq}} - 2\lambda\,, \\ 0 &= \left.\frac{\mathrm{d}\Lambda}{\mathrm{d}[\mathrm{M}^*]}\right|_{\mathrm{eq}} = \mu_{\mathrm{M}^*}^{\mathrm{eq}} - \mu_{\mathrm{W}} - \lambda = \mu_{\mathrm{M}^*}^{\circ} + RT\ln[\mathrm{M}^*]_{\mathrm{eq}} - \mu_{\mathrm{W}} - \lambda\,, \\ 0 &= \left.\frac{\mathrm{d}\Lambda}{\mathrm{d}[\mathrm{A}_2^*]}\right|_{\mathrm{eq}} = \mu_{\mathrm{A}_2^*}^{\mathrm{eq}} - 2\mu_{\mathrm{W}} - 2\lambda = \mu_{\mathrm{A}_2^*}^{\circ} + RT\ln[\mathrm{A}_2^*]_{\mathrm{eq}} - 2\mu_{\mathrm{W}} - 2\lambda\,. \end{aligned} \tag{19}$$

The equilibrium semigrand Gibbs potential reads

$$\begin{aligned} \mathcal{G}_{\mathrm{eq}} &= \lambda L_{\mathrm{M}} - RT\left([\mathrm{M}]_{\mathrm{eq}} + [\mathrm{A}_2]_{\mathrm{eq}} + [\mathrm{M}^*]_{\mathrm{eq}} + [\mathrm{A}_2^*]_{\mathrm{eq}} + [\mathrm{F}]_{\mathrm{eq}} + [\mathrm{W}]_{\mathrm{eq}}\right) \\ &= [\mathrm{M}]\mu_{\mathrm{M}}^{\mathrm{eq}} + [\mathrm{A}_2]\mu_{\mathrm{A}_2}^{\mathrm{eq}} + [\mathrm{M}^*]\left(\mu_{\mathrm{M}^*}^{\mathrm{eq}} - \mu_{\mathrm{W}}\right) + [\mathrm{A}_2^*]\left(\mu_{\mathrm{A}_2^*}^{\mathrm{eq}} - 2\mu_{\mathrm{W}}\right) + \\ &\quad - RT\left([\mathrm{M}]_{\mathrm{eq}} + [\mathrm{A}_2]_{\mathrm{eq}} + [\mathrm{M}^*]_{\mathrm{eq}} + [\mathrm{A}_2^*]_{\mathrm{eq}} + [\mathrm{F}]_{\mathrm{eq}} + [\mathrm{W}]_{\mathrm{eq}}\right)\,, \end{aligned} \tag{20}$$

which leads by direct calculation to Equation (3) in the main text:

$$\mathcal{G} - \mathcal{G}_{\mathrm{eq}} = RT\sum_X\left[[X]\ln\frac{[X]}{[X]_{\mathrm{eq}}} - [X] + [X]_{\mathrm{eq}}\right] \geq 0\,. \tag{21}$$

Therefore, when $\mu_{\mathrm{F}} = \mu_{\mathrm{W}}$, the quantity $\mathcal{G} - \mathcal{G}_{\mathrm{eq}}$ is a Lyapunov function for the open network relaxing to equilibrium. When $\mathcal{F}_{\mathrm{fuel}} = \mu_{\mathrm{F}} - \mu_{\mathrm{W}} \neq 0$, the fueling chemical work in Supplementary Equation 16 does not vanish, and the system is prevented from reaching equilibrium.

Equation (1) in the main text is obtained by integrating Supplementary Equation 16 from time $t = 0$ to a generic time t. In our simulation of energy storage, we focused on the special case in which the system at time $t = 0$ is at equilibrium ($\mathcal{F}_{\mathrm{fuel}} = 0$).

We end this section by analytically proving that η_{es} defined in the main text goes to zero both in the short and in the long time limits.

In the short time limit $t = \delta t \ll 1$, *i.e.* immediately after tuning on $\mathcal{F}_{\mathrm{fuel}}$ by changing [F], we have

$$\begin{aligned} \Delta\mathcal{G} &\simeq \mathrm{d}_t\mathcal{G}|_0\,\delta t + \mathrm{d}_t^2\mathcal{G}|_0\,\delta t^2 \\ \mathcal{W}_{\mathrm{fuel}} &\simeq \dot{\mathcal{W}}_{\mathrm{fuel}}(0)\,\delta t + \mathrm{d}_t\dot{\mathcal{W}}_{\mathrm{fuel}}|_0\,\delta t^2\,, \end{aligned} \tag{22}$$

While $\dot{\mathcal{W}}_{\mathrm{fuel}}(0) \neq 0$, $\mathrm{d}_t\mathcal{G}|_0 = 0$. Indeed, by using Supplementary Equation17, we find that

$$\mathrm{d}_t\mathcal{G}|_0 = \textstyle\sum_X \hat{\mu}_X(0)\,\mathrm{d}_t[X]|_0 = \sum_X \hat{\mu}_X(0)\,\mathbb{S}^X \boldsymbol{J}(0)\,, \tag{23}$$

where $\hat{\mu}_X$ is equal to

$$\hat{\mu}_{\mathrm{M}} = \mu_{\mathrm{M}}\,,\quad \hat{\mu}_{\mathrm{M}^*} = \mu_{\mathrm{M}^*} - \mu_{\mathrm{W}}\,,\quad \hat{\mu}_{\mathrm{A}_2^*} = \mu_{\mathrm{A}_2^*} - 2\mu_{\mathrm{W}}\,,\quad \hat{\mu}_{\mathrm{A}_2} = \mu_{\mathrm{A}_2}\,. \tag{24}$$

At $t = 0$, the concentrations of internal species X as well as their chemical potentials μ_X are at equilibrium. By using Supplementary Equation 19, one readily sees that $\sum_X \hat{\mu}_X^{\mathrm{eq}}\,\mathbb{S}^X = 0$ for all reactions, and from Supplementary Equation 23 one proves that $\mathrm{d}_t\mathcal{G}|_0 = 0$. Therefore

$$\eta_{\mathrm{es}}(\delta t) \simeq \frac{\mathrm{d}_t^2\mathcal{G}|_0\,\delta t}{\dot{\mathcal{W}}_{\mathrm{fuel}}(0)} \tag{25}$$

goes to zero when δt goes to zero.

In the long time limit $t \to \infty$, the system approaches a steady state in which $\mathrm{d}_t \mathcal{G} \to 0$, and thus $\dot{\mathcal{W}}_{\text{fuel}} \simeq \dot{\Sigma} \geq 0$. Therefore, while $\Delta\mathcal{G}$ remains finite, $\mathcal{W}_{\text{fuel}}$ keeps growing, and $\eta_{\text{es}} \to 0$.

Since in ES $\Delta\mathcal{G} \geq 0$, as announced, η_{es} will start in zero, increase in time, reach a maximum, and then eventually decrease back to zero. The above proof can be generalized to arbitrary chemical reaction networks evolving towards steady states, but η_{es} might have more than one local maximum depending on the underlying dynamics.

c. Cycles & kinetic symmetry

From the thermodynamic point of view we adopted in this work, any steady state other than the equilibrium one has a non null energy content which is quantified through its concentrations distribution according to Supplementary Equation 21 (equation (3) in the main text). A condition referred to as "kinetic symmetry" is central in the literature on ES [4, 5]. This section aims to frame this concept into our theory.

In a CRN, a *cycle* is a reaction pathway which does not alter the internal state of the system. They play an important role at steady state, where chemical currents can only flow along cycles. Any possible cycle is represented by a vector in the kernel of $\mathbb{S}^{\text{X}}$, which is spanned by

$$\boldsymbol{c}_1^{\text{T}} = \begin{array}{c} \scriptstyle{1\text{F} \quad 1\text{W} \quad 2 \quad 3\text{F} \quad 3\text{W} \quad 4} \\ \begin{pmatrix} 1 & -1 & 0 & 0 & 0 & 0 \end{pmatrix} \end{array}, \tag{26}$$

$$\boldsymbol{c}_2^{\text{T}} = \begin{array}{c} \scriptstyle{1\text{F} \quad 1\text{W} \quad 2 \quad 3\text{F} \quad 3\text{W} \quad 4} \\ \begin{pmatrix} 0 & 0 & 0 & 1 & -1 & 0 \end{pmatrix} \end{array}, \tag{27}$$

$$\boldsymbol{c}_3^{\text{T}} = \begin{array}{c} \scriptstyle{1\text{F} \quad 1\text{W} \quad 2 \quad 3\text{F} \quad 3\text{W} \quad 4} \\ \begin{pmatrix} 2 & 0 & 1 & 0 & 1 & 1 \end{pmatrix} \end{array}, \tag{28}$$

where each entry represents the number of times the corresponding reaction has to be performed in order to complete the cycle. The vector of steady-state currents can always be expressed in terms of a complete base of cycles:

$$\boldsymbol{J} = J^{(c_1)}\boldsymbol{c}_1 + J^{(c_2)}\boldsymbol{c}_2 + J^{(c_3)}\boldsymbol{c}_3 \tag{29}$$

where the coefficients are called *cycle currents* and represent the contribution of each cycle to the total current observed along each reaction. An important thing to note is that the relation $\frac{1}{2}(J_{1\text{F}} + J_{1\text{W}}) = J_2 = J_{3\text{F}} + J_{3\text{W}} = J_4$ always holds. It shows that the net current from M to M* has to be twice as much those across other steps at the stationary state, as represented through arrow thickness in Figure 1b of the main text. When the cycle current for a particular cycle is equal to zero, we refer to that cycle as being *stalled* [6, 7].

Kinetic symmetry as defined in Supplementary Reference [5] corresponds to the situation where no accumulation of A_2 occurs in the system, i.e. when no net current from monomers to assemblies can occur. This corresponds to the situation where the cycle $\boldsymbol{c}_3$ is stalled, i.e. when $J^{(c_3)}\boldsymbol{c}_3 = 0$ in Supplementary Equation 29. Mathematically it implies

$$\frac{([\text{F}] \cdot k_{+1\text{F}} + [\text{W}] \cdot k_{+1\text{W}})^2 \, k_{+2} \, (k_{+3\text{F}} + k_{+3\text{W}}) \, k_{+4}}{(k_{-1\text{F}} + k_{-1\text{W}})^2 \, k_{-2} \, ([\text{F}]^2 \cdot k_{-3\text{F}} + [\text{W}]^2 \cdot k_{-3\text{W}}) \, k_{-4}} = 1 \,, \tag{30}$$

which is the same as Equation (2) reported in Supplementary Reference [5], but with the dependence on the chemostatted species made explicit. The above equation has two solutions in [F]. One always exists and corresponds to the equilibrium state, where by definition all the cycles are stalled and no energy is stored in the system. The other one, when physical (it may be negative), corresponds to a

nonequilibrium steady state where A_2 does not accumulate (current will instead flow along cycles $\boldsymbol{c}_1$ and $\boldsymbol{c}_2$) but ES nevertheless occurs via an increase of $\mathcal{G}$. Therefore, ES involving the accumulation of A_2 requires to break the kinetic symmetry of the network, as happens when varying [F]. We note that for the choice of kinetic constants and [W] we adopted in the paper (see Supplementary Note 1d below), the condition of nonequilibrium kinetic symmetry can't be realized with any value of $\mathcal{F}_{\text{fuel}}$ different from the equilibrium one.

d. Parameters

With reference to the model in Figure 1 of the main text, the following parameters were used for all the simulations:

Supplementary Table 1. Parameters used for the energy storage model depicted in Figure 1 of the main text. Values of the backward kinetic constants were obtain through Supplementary Equation 4 in order to assure thermodynamic consistency, here they are reported with 3 digits. For the sake of completeness, equilibrium constants of the various reactions ($K_\rho = k_{+\rho}/k_{-\rho}$) are reported. Note that [W] is kept fixed, while we used [F] to tune $\mathcal{F}_{\text{fuel}}$ in the various discussions (see Supplementary Figure 1 below).

μ°_{M}	$-2 \cdot 10^3$ J mol^{-1}
$\mu^\circ_{\text{M}^*}$	$-3 \cdot 10^3$ J mol^{-1}
$\mu^\circ_{\text{A}_2^*}$	$-4 \cdot 10^3$ J mol^{-1}
$\mu^\circ_{\text{A}_2}$	$9 \cdot 10^3$ J mol^{-1}
μ°_{F}	$11 \cdot 10^3$ J mol^{-1}
μ°_{W}	$-11 \cdot 10^3$ J mol^{-1}
L_{M}	1 M
[F]	$\left[1 \cdot 10^{-4}, 4 \cdot 10^{2}\right]$ M
[W]	1 M

$k_{+1\text{F}}$	5 M^{-1}s^{-1}
$k_{+1\text{W}}$	$1 \cdot 10^{-3}$ M^{-1}s^{-1}
k_{+2}	1 M^{-1}s^{-1}
$k_{+3\text{F}}$	$1 \cdot 10^{-6}$ s^{-1}
$k_{+3\text{W}}$	5 s^{-1}
k_{+4}	$1 \cdot 10^{-1}$ s^{-1}
$k_{-1\text{F}}$	$3.63 \cdot 10^{-2}$ s^{-1}
$k_{-1\text{W}}$	$6.06 \cdot 10^{-2}$ s^{-1}
k_{-2}	2.27 s^{-1}
$k_{-3\text{F}}$	1.74 M^{-2}s^{-1}
$k_{-3\text{W}}$	$1.24 \cdot 10^{-1}$ M^{-2}s^{-1}
k_{-4}	$4.81 \cdot 10^{-4}$ M^{-1}s^{-1}

$K_{1\text{F}}$	$1.38 \cdot 10^{2}$ M^{-1}
$K_{1\text{W}}$	$1.65 \cdot 10^{-2}$ M^{-1}
K_2	$4.40 \cdot 10^{-1}$ M^{-1}
$K_{3\text{F}}$	$5.76 \cdot 10^{-7}$ M^2
$K_{3\text{W}}$	$4.02 \cdot 10^{1}$ M^2
K_4	$2.08 \cdot 10^{2}$ M

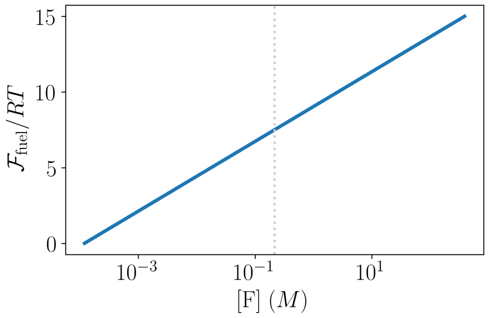

Supplementary Figure 1. Values of $\mathcal{F}_{\text{fuel}}$ as a function of [F] (note the logarithmic scale for the x axis). The value of [F] giving $\mathcal{F}_{\text{fuel}} = 7.5 \cdot RT$ is highlighted by the vertical dotted line.

SUPPLEMENTARY NOTE 2: DETAILS ON DRIVEN SYNTHESIS

a. Dynamics

With the addition of the extraction mechanism, the evolution in time of the concentrations of the species M, M*, A_2, and A_2^* is ruled by the following rate equations

$$\mathrm{d}_t \underbrace{\begin{pmatrix}[\mathrm{M}]\\ [\mathrm{M}^*]\\ [\mathrm{A}_2^*]\\ [\mathrm{A}_2]\end{pmatrix}}_{[\boldsymbol{X}]} = \underbrace{\begin{pmatrix}-1 & -1 & 0 & 0 & 0 & 2\\ 1 & 1 & -2 & 0 & 0 & 0\\ 0 & 0 & 1 & -1 & -1 & 0\\ 0 & 0 & 0 & 1 & 1 & -1\end{pmatrix}}_{\mathbb{S}^{\mathrm{X}}} \cdot \underbrace{\begin{pmatrix}k_{+1\mathrm{F}}[\mathrm{F}][\mathrm{M}] - k_{-1\mathrm{F}}[\mathrm{M}^*]\\ k_{+1\mathrm{W}}[\mathrm{W}][\mathrm{M}] - k_{-1\mathrm{W}}[\mathrm{M}^*]\\ k_{+2}[\mathrm{M}^*]^2 - k_{-2}[\mathrm{A}_2^*]\\ k_{+3\mathrm{F}}[\mathrm{A}_2^*] - k_{-3\mathrm{F}}[\mathrm{A}_2][\mathrm{F}]^2\\ k_{+3\mathrm{W}}[\mathrm{A}_2^*] - k_{-3\mathrm{W}}[\mathrm{A}_2][\mathrm{W}]^2\\ k_{+4}[\mathrm{A}_2] - k_{-4}[\mathrm{M}]^2\end{pmatrix}}_{\boldsymbol{J} = \boldsymbol{J}_+ - \boldsymbol{J}_-} + \begin{pmatrix}2I_{\mathrm{ext}}\\ 0\\ 0\\ -I_{\mathrm{ext}}\end{pmatrix}, \tag{31}$$

where the current of extraction reads $I_{\mathrm{ext}} = k_{\mathrm{ext}}[\mathrm{A}_2]$.

We examine this system at the steady state, in which all concentrations are stationary: $\mathrm{d}_t[X]_{\mathrm{ss}} = 0$ for all X. Their expressions are not analytical, but can be easily obtained numerically, thus showing that the steady state state is unique within a broad range of values for the parameters that we examined.

b. Thermodynamics

For the driven synthesis model at the steady state, Supplementary Equation 8 becomes

$$T\dot{\Sigma} = \dot{\mathcal{W}}_{\mathrm{chem}}\,, \tag{32}$$

where the chemical work per unit of time now reads

$$\dot{\mathcal{W}}_{\mathrm{chem}} = \mu_{\mathrm{F}} I_{\mathrm{F}} + \mu_{\mathrm{W}} I_{\mathrm{W}} + 2\mu_{\mathrm{M}} I_{\mathrm{ext}} - \mu_{\mathrm{A}_2} I_{\mathrm{ext}}\,. \tag{33}$$

In order to construct the entropy balance as in Equation (4) of the main text, we once again need to consider conservation vectors (11) and (12), *i.e.* a basis of the cokernel of $\mathbb{S}$.

$$\boldsymbol{\ell}_{\mathrm{M}} = \begin{matrix} \scriptstyle\mathrm{M} & \scriptstyle\mathrm{M}^* & \scriptstyle\mathrm{A}_2^* & \scriptstyle\mathrm{A}_2 & \scriptstyle\mathrm{F} & \scriptstyle\mathrm{W} \\ (\,1 & 1 & 2 & 2 & 0 & 0\,) \end{matrix}, \tag{34}$$

$$\boldsymbol{\ell}_{\mathrm{W}} = \begin{matrix} \scriptstyle\mathrm{M} & \scriptstyle\mathrm{M}^* & \scriptstyle\mathrm{A}_2^* & \scriptstyle\mathrm{A}_2 & \scriptstyle\mathrm{F} & \scriptstyle\mathrm{W} \\ (\,0 & 1 & 2 & 0 & 1 & 1\,) \end{matrix}. \tag{35}$$

Now, both these vectors identify broken conserved quantities. The former corresponds to the conserved quantity relative to the monomer

$$L_{\mathrm{M}} = \boldsymbol{\ell}_{\mathrm{M}} \cdot \begin{pmatrix}[\boldsymbol{X}]\\ [\boldsymbol{Y}]\end{pmatrix} = [\mathrm{M}] + [\mathrm{M}^*] + 2[\mathrm{A}_2^*] + 2[\mathrm{A}_2]\,. \tag{36}$$

In the framework of Supplementary Reference [1], this is a broken conservation law because of the presence of the extraction mechanism. Here its value does not change by construction of the model, since every A_2 which is exchanged is readily replaced by 2 M molecules

$$\mathrm{d}_t L_{\mathrm{M}} = 2I_{\mathrm{ext}} - 2I_{\mathrm{ext}} = 0\,. \tag{37}$$

The latter represents the F/W conservation law

$$L_{\mathrm{W}} = \boldsymbol{\ell}_{\mathrm{W}} \cdot \begin{pmatrix} [\boldsymbol{X}] \\ [\boldsymbol{Y}] \end{pmatrix} = [\mathrm{M}^*] + 2[\mathrm{A}_2^*] + [\mathrm{F}] + [\mathrm{W}]\,, \tag{38}$$

which is broken by the fueling mechanism

$$\mathrm{d}_t L_{\mathrm{W}} = I_{\mathrm{F}} + I_{\mathrm{W}}\,. \tag{39}$$

At the steady state all time derivative vanish, and we can use Supplementary Equation 39 to recast the chemical work per unit of time in Supplementary Equation 33 into

$$\dot{\mathcal{W}}_{\mathrm{chem}} = \dot{\mathcal{W}}_{\mathrm{fuel}} + \dot{\mathcal{W}}_{\mathrm{ext}} \tag{40}$$

where

$$\dot{\mathcal{W}}_{\mathrm{fuel}} = I_{\mathrm{F}}\left(\mu_{\mathrm{F}} - \mu_{\mathrm{W}}\right)\,. \tag{41}$$

is the input power, and

$$\dot{\mathcal{W}}_{\mathrm{ext}} = I_{\mathrm{ext}}(2\mu_{\mathrm{M}} - \mu_{\mathrm{A}_2}) \tag{42}$$

is the output power. By combining Supplementary Equation 40 with Supplementary Equation 32, we obtain Equation (4) of the main text.

c. Plots of $-\dot{\mathcal{W}}_{\mathrm{ext}}$ and $\dot{\mathcal{W}}_{\mathrm{fuel}}$

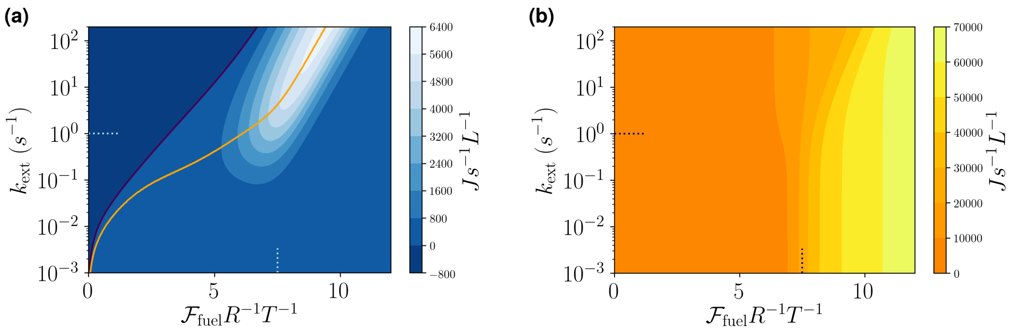

Supplementary Figure 2. **(a)** Minus the output power ($-\dot{\mathcal{W}}_{\mathrm{ext}}$) and **(b)** input power ($\dot{\mathcal{W}}_{\mathrm{fuel}}$) plotted in the same range of parameter as in Figure 4 of the main text. The efficiency is given by the ratio of the two plots, according to Equation (5) of the main text.

d. Linear Regime

For $k_{\mathrm{ext}} = 0$ and $\mathcal{F}_{\mathrm{fuel}} = \mu_{\mathrm{F}} - \mu_{\mathrm{W}} = 0$, the entropy production at the steady state vanishes, and hence the steady state is an equilibrium one ($[X]_{\mathrm{eq}}$). For

$$k_{\mathrm{ext}} \ll 1 \tag{43a}$$

$$\mathcal{F}_{\mathrm{fuel}} = \mu_{\mathrm{F}} - \mu_{\mathrm{W}} \ll RT \tag{43b}$$

the entropy production is close to zero and hence the system is in a linear regime close to equilibrium. In this regime, we can write the steady-state concentrations as $[X]_{\mathrm{ss}} = [X]_{\mathrm{eq}}(1 + f_X/RT)$, where $f_X \ll RT$ for all X encode the linear shifts from equilibrium. Regarding the chemostatted ones, without loss of generality, we write $[\mathrm{F}] = [\mathrm{F}]_{\mathrm{eq}}(1 + \mathcal{F}_{\mathrm{fuel}}/RT)$ and $[\mathrm{W}] = [\mathrm{W}]_{\mathrm{eq}}$, where $\mu^{\circ}_{\mathrm{F}} + RT\ln[\mathrm{F}]_{\mathrm{eq}} = \mu^{\circ}_{\mathrm{W}} + RT\ln[\mathrm{W}]_{\mathrm{eq}}$. In this way, when approximating the chemical potentials of the chemostats using the fact that $\mathcal{F}_{\mathrm{fuel}} \ll RT$, the equality in Supplementary Equation 43b is recovered.

By inserting the above expressions into the rate equations, Supplementary Equation 31 and 2, one obtains the analytical solution of the driven synthesis model at the steady-state in the linear regime. Indeed, by discarding second order terms and exploiting the properties of the equilibrium state ($J^{\mathrm{eq}}_{+\rho} = J^{\mathrm{eq}}_{-\rho}$), the rate equations read

$$\mathbb{M}^{\mathrm{X}}_{\mathrm{X}} \cdot \begin{pmatrix} f_{\mathrm{M}} \\ f_{\mathrm{M}^*} \\ f_{\mathrm{A}_2^*} \\ f_{\mathrm{A}_2} \end{pmatrix} + \mathcal{F}_{\mathrm{fuel}}\, \mathbb{M}^{\mathrm{X}}_{\mathrm{F}} = \begin{pmatrix} 2I_{\mathrm{ext}} \\ 0 \\ 0 \\ -I_{\mathrm{ext}} \end{pmatrix} \qquad \text{and} \qquad \mathbb{M}^{\mathrm{F}}_{\mathrm{X}} \cdot \begin{pmatrix} f_{\mathrm{M}} \\ f_{\mathrm{M}^*} \\ f_{\mathrm{A}_2^*} \\ f_{\mathrm{A}_2} \end{pmatrix} + \mathcal{F}_{\mathrm{fuel}}\, \mathbb{M}^{\mathrm{F}}_{\mathrm{F}} = I_{\mathrm{F}}\,, \tag{44}$$

for the internal and chemostatted species, respectively. The extraction current is given by $I_{\mathrm{ext}} = k_{\mathrm{ext}}[\mathrm{A}_2]_{\mathrm{eq}}$, while the matrix $\mathbb{M}$ is a 6 by 6 matrix which encodes both the topology and the kinetics of the linear regime dynamics

$$\mathbb{M} := \mathbb{S} \cdot \mathrm{diag}\left\{ \boldsymbol{J}^{\mathrm{eq}}_{+} \right\} \cdot \mathbb{S}^{\mathsf{T}}/RT\,, \tag{45}$$

where

$$\boldsymbol{J}^{\mathrm{eq}}_{+} = \left(k_{+1\mathrm{F}}[\mathrm{F}]_{\mathrm{eq}}[\mathrm{M}]_{\mathrm{eq}} \quad k_{+1\mathrm{W}}[\mathrm{W}]_{\mathrm{eq}}[\mathrm{M}]_{\mathrm{eq}} \quad k_{+2}[\mathrm{M}^*]^2_{\mathrm{eq}} \quad k_{+3\mathrm{F}}[\mathrm{A}_2^*]_{\mathrm{eq}} \quad k_{+3\mathrm{W}}[\mathrm{A}_2^*]_{\mathrm{eq}} \quad k_{+4}[\mathrm{A}_2]_{\mathrm{eq}} \right) \tag{46}$$

are the equilibrium forward fluxes. The labels X and F in Supplementary Equation 44 select blocks of $\mathbb{M}$ corresponding to internal and fuel species, respectively, as shown below.

$$\mathbb{M} = \begin{array}{c} \\ \mathrm{M} \\ \mathrm{M}^* \\ \mathrm{A}_2 \\ \mathrm{A}_2^* \\ \mathrm{F} \\ \mathrm{W} \end{array} \overset{\begin{array}{cccccc} \mathrm{M} & \mathrm{M}^* & \mathrm{A}_2^* & \mathrm{A}_2 & \mathrm{F} & \mathrm{W} \end{array}}{\left[\begin{array}{c|c|c} \mathbb{M}^{\mathrm{X}}_{\mathrm{X}} & \mathbb{M}^{\mathrm{X}}_{\mathrm{F}} & \mathbb{M}^{\mathrm{X}}_{\mathrm{W}} \\ \hline \mathbb{M}^{\mathrm{F}}_{\mathrm{X}} & \multicolumn{2}{c}{} \\ \cline{1-1} \mathbb{M}^{\mathrm{W}}_{\mathrm{X}} & \multicolumn{2}{c}{\mathbb{M}^{\mathrm{F,W}}_{\mathrm{F,W}}} \end{array} \right]} \tag{47}$$

Let us now introduce the index "a" to denote the activated species which are neither exchanged nor extracted (M^* and A_2^*), whereas the index "e" denotes the extracted/injected species (A_2 and M). The rate equations can thus be further split into

$$\begin{aligned} 0 &= \mathcal{F}_{\mathrm{fuel}}\, \mathbb{M}^{\mathrm{a}}_{\mathrm{F}} + \mathbb{M}^{\mathrm{a}}_{\mathrm{a}} \cdot \begin{pmatrix} f_{\mathrm{M}^*} \\ f_{\mathrm{A}_2^*} \end{pmatrix} + \mathbb{M}^{\mathrm{a}}_{\mathrm{e}} \cdot \begin{pmatrix} f_{\mathrm{M}} \\ f_{\mathrm{A}_2} \end{pmatrix} \\ -I_{\mathrm{ext}} &= \mathcal{F}_{\mathrm{fuel}}\, \mathbb{M}^{\mathrm{A}_2}_{\mathrm{F}} + \mathbb{M}^{\mathrm{A}_2}_{\mathrm{a}} \cdot \begin{pmatrix} f_{\mathrm{M}^*} \\ f_{\mathrm{A}_2^*} \end{pmatrix} + \mathbb{M}^{\mathrm{A}_2}_{\mathrm{e}} \cdot \begin{pmatrix} f_{\mathrm{M}} \\ f_{\mathrm{A}_2} \end{pmatrix} \\ I_{\mathrm{F}} &= \mathcal{F}_{\mathrm{fuel}}\, \mathbb{M}^{\mathrm{F}}_{\mathrm{F}} + \mathbb{M}^{\mathrm{F}}_{\mathrm{a}} \cdot \begin{pmatrix} f_{\mathrm{M}^*} \\ f_{\mathrm{A}_2^*} \end{pmatrix} + \mathbb{M}^{\mathrm{F}}_{\mathrm{e}} \cdot \begin{pmatrix} f_{\mathrm{M}} \\ f_{\mathrm{A}_2} \end{pmatrix}\,. \end{aligned} \tag{48}$$

We now observe that from the definition of conservation law, the following constraint holds

$$\mathbf{0} = \mathbb{M}\,\boldsymbol{\ell}_{\mathrm{M}}^{\mathrm{T}} = \mathbb{M}_{\mathrm{a}} \cdot \begin{pmatrix} 1 \\ 2 \end{pmatrix} + \mathbb{M}_{\mathrm{e}} \cdot \begin{pmatrix} 1 \\ 2 \end{pmatrix} , \tag{49}$$

which implies that

$$\mathbb{M}_{\mathrm{M}} = -2\mathbb{M}_{\mathrm{A}_2} - \mathbb{M}_{\mathrm{a}} \cdot \begin{pmatrix} 1 \\ 2 \end{pmatrix} . \tag{50}$$

This allows us to rewrite Supplementary Equations 48 as

$$\begin{aligned} 0 &= \mathcal{F}_{\mathrm{fuel}}\, \mathbb{M}_{\mathrm{F}}^{\mathrm{a}} + \mathbb{M}_{\mathrm{a}}^{\mathrm{a}} \cdot \begin{pmatrix} f_{\mathrm{M}^*} - f_{\mathrm{M}} \\ f_{\mathrm{A}_2^*} - 2f_{\mathrm{M}} \end{pmatrix} + \left(f_{\mathrm{A}_2} - 2f_{\mathrm{M}}\right)\, \mathbb{M}_{\mathrm{A}_2}^{\mathrm{a}} \\ -I_{\mathrm{ext}} &= \mathbb{M}_{\mathrm{F}}^{\mathrm{A}_2}\, \mathcal{F}_{\mathrm{fuel}} + \mathbb{M}_{\mathrm{a}}^{\mathrm{A}_2} \cdot \begin{pmatrix} f_{\mathrm{M}^*} - f_{\mathrm{M}} \\ f_{\mathrm{A}_2^*} - 2f_{\mathrm{M}} \end{pmatrix} + \left(f_{\mathrm{A}_2} - 2f_{\mathrm{M}}\right)\, \mathbb{M}_{\mathrm{A}_2}^{\mathrm{A}_2} \\ I_{\mathrm{F}} &= \mathcal{F}_{\mathrm{fuel}}\, \mathbb{M}_{\mathrm{F}}^{\mathrm{F}} + \mathbb{M}_{\mathrm{a}}^{\mathrm{F}} \cdot \begin{pmatrix} f_{\mathrm{M}^*} - f_{\mathrm{M}} \\ f_{\mathrm{A}_2^*} - 2f_{\mathrm{M}} \end{pmatrix} + \left(f_{\mathrm{A}_2} - 2f_{\mathrm{M}}\right)\, \mathbb{M}_{\mathrm{A}_2}^{\mathrm{F}} . \end{aligned} \tag{51}$$

We now solve the first of the three equations above for the vector in parenthesis, using the fact that $\mathbb{M}_{\mathrm{a}}^{\mathrm{a}}$ is nonsingular.

$$\begin{pmatrix} f_{\mathrm{M}^*} - f_{\mathrm{M}} \\ f_{\mathrm{A}_2^*} - 2f_{\mathrm{M}} \end{pmatrix} = -(\mathbb{M}_{\mathrm{a}}^{\mathrm{a}})^{-1} \cdot \left[\mathcal{F}_{\mathrm{fuel}}\, \mathbb{M}_{\mathrm{F}}^{\mathrm{a}} + \left(f_{\mathrm{A}_2} - 2f_{\mathrm{M}}\right)\, \mathbb{M}_{\mathrm{A}_2}^{\mathrm{a}}\right] . \tag{52}$$

This follows from the fact that $\mathbb{M}_{\mathrm{a}}^{\mathrm{a}}$ is Gramian [8], and $\mathbb{S}^{\mathrm{a}}$ contains linearly independent vectors. Therefore, the last two equations in 51 can be recast into

$$\begin{aligned} -I_{\mathrm{ext}} &= \mathcal{F}_{\mathrm{fuel}} \left[\mathbb{M}_{\mathrm{F}}^{\mathrm{A}_2} - \mathbb{M}_{\mathrm{a}}^{\mathrm{A}_2} \cdot (\mathbb{M}_{\mathrm{a}}^{\mathrm{a}})^{-1} \cdot \mathbb{M}_{\mathrm{F}}^{\mathrm{a}}\right] + \left(f_{\mathrm{A}_2} - 2f_{\mathrm{M}}\right) \left[\mathbb{M}_{\mathrm{A}_2}^{\mathrm{A}_2} - \mathbb{M}_{\mathrm{a}}^{\mathrm{A}_2} \cdot (\mathbb{M}_{\mathrm{a}}^{\mathrm{a}})^{-1} \cdot \mathbb{M}_{\mathrm{A}_2}^{\mathrm{a}}\right] \\ I_{\mathrm{F}} &= \mathcal{F}_{\mathrm{fuel}} \left[\mathbb{M}_{\mathrm{F}}^{\mathrm{F}} - \mathbb{M}_{\mathrm{a}}^{\mathrm{F}} \cdot (\mathbb{M}_{\mathrm{a}}^{\mathrm{a}})^{-1} \cdot \mathbb{M}_{\mathrm{F}}^{\mathrm{a}}\right] + \left(f_{\mathrm{A}_2} - 2f_{\mathrm{M}}\right) \left[\mathbb{M}_{\mathrm{A}_2}^{\mathrm{F}} - \mathbb{M}_{\mathrm{a}}^{\mathrm{F}} \cdot (\mathbb{M}_{\mathrm{a}}^{\mathrm{a}})^{-1} \cdot \mathbb{M}_{\mathrm{A}_2}^{\mathrm{a}}\right] . \end{aligned} \tag{53}$$

Changing signs conveniently, we can rewrite the above equations in terms of the Onsager matrix $\mathbb{L}$, which expresses the linear dependence of currents from forces when the system is close to equilibrium:

$$\begin{pmatrix} I_F \\ I_{\mathrm{ext}} \end{pmatrix} = \mathbb{L} \begin{pmatrix} \mu_{\mathrm{F}} - \mu_{\mathrm{W}} \\ 2\mu_{\mathrm{M}} - \mu_{\mathrm{A}_2} \end{pmatrix} . \tag{54}$$

Indeed, in the linear regime the chemical force associated to the extraction currents is $2\mu_{\mathrm{M}} - \mu_{\mathrm{A}_2} = 2f_{\mathrm{M}} - f_{\mathrm{A}_2}$. The entries of the Onsager matrix are given by

$$\mathbb{L} = \begin{pmatrix} \mathbb{M}_{\mathrm{F}}^{\mathrm{F}} - \mathbb{M}_{\mathrm{a}}^{\mathrm{F}} \cdot (\mathbb{M}_{\mathrm{a}}^{\mathrm{a}})^{-1} \cdot \mathbb{M}_{\mathrm{F}}^{\mathrm{a}} & \mathbb{M}_{\mathrm{a}}^{\mathrm{F}} \cdot (\mathbb{M}_{\mathrm{a}}^{\mathrm{a}})^{-1} \cdot \mathbb{M}_{\mathrm{A}_2}^{\mathrm{a}} - \mathbb{M}_{\mathrm{A}_2}^{\mathrm{F}} \\ \mathbb{M}_{\mathrm{A}_2}^{\mathrm{A}_2} - \mathbb{M}_{\mathrm{a}}^{\mathrm{A}_2} \cdot (\mathbb{M}_{\mathrm{a}}^{\mathrm{a}})^{-1} \cdot \mathbb{M}_{\mathrm{A}_2}^{\mathrm{a}} & \mathbb{M}_{\mathrm{a}}^{\mathrm{A}_2} \cdot (\mathbb{M}_{\mathrm{a}}^{\mathrm{a}})^{-1} \cdot \mathbb{M}_{\mathrm{F}}^{\mathrm{a}} - \mathbb{M}_{\mathrm{F}}^{\mathrm{A}_2} \end{pmatrix} := \begin{pmatrix} \mathbb{L}_{11} & \mathbb{L}_{12} \\ \mathbb{L}_{21} & \mathbb{L}_{22} \end{pmatrix} . \tag{55}$$

We can use Supplementary Equation 54 to analytically evaluate the efficiency η_{ds} introduced in Equation (5) of the main text, as well as the output power $\dot{\mathcal{W}}_{\mathrm{ext}}$, in terms of k_{ext} and $\mathcal{F}_{\mathrm{fuel}}$, namely the control parameters in the model:

$$\eta_{\mathrm{ds}} = -\frac{I_{\mathrm{ext}}(I_{\mathrm{ext}} - \mathcal{F}_{\mathrm{fuel}}\mathbb{L}_{12})}{\mathcal{F}_{\mathrm{fuel}}(I_{\mathrm{ext}}\mathbb{L}_{12} + \mathcal{F}_{\mathrm{fuel}}\det[\mathbb{L}])} ; \quad \dot{\mathcal{W}}_{\mathrm{ext}} = \frac{I_{\mathrm{ext}}(I_{\mathrm{ext}} - \mathcal{F}_{\mathrm{fuel}}\mathbb{L}_{12})}{\mathbb{L}_{11}} . \tag{56}$$

When $\mathcal{F}_{\rm fuel}$ is kept fixed, the values of $k_{\rm ext}$ which extremise $\eta_{\rm ds}$ and $-\dot{\mathcal{W}}_{\rm ext}$ are readily found by deriving the previous expressions and look for the unique stable points:

$$\text{max efficiency}: \quad k^*_{\rm ext} = \frac{\sqrt{\mathbb{L}_{11}\mathbb{L}_{22}\det[\mathbb{L}]} - \det[\mathbb{L}]}{\mathbb{L}_{12}[\mathrm{A}_2]_{\rm eq}}\mathcal{F}_{\rm fuel} \tag{57}$$

$$\text{max output power}: \quad k^*_{\rm ext} = \frac{\mathbb{L}_{12}}{2[\mathrm{A}_2]_{\rm eq}}\mathcal{F}_{\rm fuel} \,. \tag{58}$$

The above equations define the sets of points of maximum efficiency and efficiency at maximum power for any value of $\mathcal{F}_{\rm fuel}$ within the linear regime. By equating the right hand sides of Supplementary Equations 57 and 58, one obtains that these two expressions coicide if and only if $\mathbb{L}_{12} = \mathbb{L}_{21} = 0$, which is never the case for coupled currents.

When evaluated using the parameters in Supplementary Table 1, Supplementary Equation 55 reads

$$\mathbb{L} = \begin{pmatrix} 17.7835 & 3.74893 \\ 3.74893 & 23.7732 \end{pmatrix} \cdot 10^{-8}\, mol^2/sLJ \tag{59}$$

where the cross coefficients are equal according to the Onsager reciprocal relations.

When the analytical solution is plotted against $k_{\rm ext}$ and $\mathcal{F}_{\rm fuel}$, we obtain the plot in Supplementary Figure 3b, where both maximum efficiency and efficiency at maximum power are highlighted as in Figure 3 of the main text. An enlargement of the linear region of Figure 3 of the main text is shown in Supplementary Figure 3a.

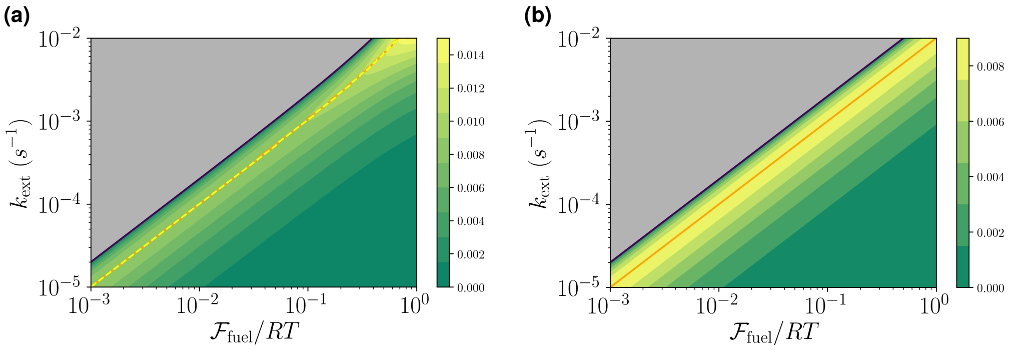

Supplementary Figure 3. Comparison between exact simulation of the full dynamics **(a)** and analytical formula obtained in the linear regime **(b)** for the efficiency in the linear regime. The log scale emphasizes the changes of magnitude of these values. For low forces and extraction rates — where Supplementary Equation 54 is a good approximation — the two plots clearly coincide. When $\mathcal{F}_{\rm fuel}$ is of the order of 0.1 (in units of RT) and $k_{\rm ext}$ reaches $10^{-3}\, s^{-1}$ differences in both numerical values and shape emerge. In particular, we see that the increase in efficiency visible for high $\mathcal{F}_{\rm fuel}$ and $k_{\rm ext}$ in (a) is a genuine nonequilibrium feature as it is absent in the linear regime, (b).

SUPPLEMENTARY REFERENCES

[1] Riccardo Rao and Massimiliano Esposito, "Nonequilibrium thermodynamics of chemical reaction networks: Wisdom from stochastic thermodynamics," Phys. Rev. X **6**, 041064 (2016).
[2] Gianmaria Falasco, Riccardo Rao, and Massimiliano Esposito, "Information thermodynamics of turing patterns," Phys. Rev. Lett. **121**, 108301 (2018).
[3] Riccardo Rao and Massimiliano Esposito, "Conservation laws and work fluctuation relations in chemical reaction networks," J. Chem. Phys. **149**, 245101 (2018).

[4] R.D. Astumian, “Stochastic pumping of non-equilibrium steady-states: how molecules adapt to a fluctuating environment,” ChemComm **54**, 427–444 (2018).

[5] G. Ragazzon and L. J. Prins, “Energy consumption in chemical fuel-driven self-assembly,” Nat. Nanotechnol. **13**, 882–889 (2018).

[6] B. Altaner, M. Polettini, and M. Esposito, “Fluctuation-dissipation relations far from equilibrium,” Phys. Rev. Lett. **117**, 180601 (2016).

[7] Matteo Polettini and Massimiliano Esposito, “Effective fluctuation and response theory,” J. Stat. Phys. (2019), 10.1007/s10955-019-02291-7.

[8] R.A. Horn and C.R. Johnson, *Matrix Analysis* (Cambridge University Press, 1985).
